# Effects of Episodic Food Insecurity on Psychological and Physiological Responses in African American Women With Obesity (RESPONSES): Protocol for a Longitudinal Observational Cohort Study

**DOI:** 10.2196/52193

**Published:** 2023-12-20

**Authors:** Candice A Myers, Robbie A Beyl, Daniel S Hsia, Melissa N Harris, Isabella J Reed, Danielle D Eliser, Lauren Bagneris, John W Apolzan

**Affiliations:** 1 Pennington Biomedical Research Center Baton Rouge, LA United States; 2 Louisiana State University Health Sciences Center New Orleans, LA United States

**Keywords:** food security, body weight, racially minoritized group, low income, stress, cortisol, allostatic load

## Abstract

**Background:**

Food insecurity is a risk factor for multiple chronic diseases, including obesity. Importantly, both food insecurity and obesity are more prevalent in African American women than in other groups. Furthermore, food insecurity is considered a *cyclic phenomenon*, with episodes of food adequacy (ie, enough food to eat) and food shortage (ie, not enough food to eat). More research is needed to better understand why food insecurity is linked to obesity, including acknowledging the episodic nature of food insecurity as a stressor and identifying underlying mechanisms.

**Objective:**

The objective of this study is to investigate the episodic nature of food insecurity as a stressor via responses in body weight and psychological and physiological parameters longitudinally and do so in a health-disparate population—African American women.

**Methods:**

We enrolled 60 African American women (food-insecure cohort: n=30, 50%; food-secure cohort: n=30, 50%) aged 18-65 years with obesity (BMI 30-50 kg/m^2^) to measure (1) daily body weight remotely over 22 weeks and (2) psychological and physiological parameters via clinic assessments at the beginning and end of the 22-week study. Furthermore, we are assessing episodes of food insecurity, stress, hedonic eating, and appetite on a weekly basis. We hypothesize that food-insecure African American women with obesity will demonstrate increased body weight and changes in psychological and physiological end points, whereas food-secure African American women with obesity will not. We are also examining associations between changes in psychological and physiological parameters and changes in body weight and performing a mediation analysis on the psychological parameters assessed at the study midpoint. Psychological questionnaires are used to assess stress; executive function, decision-making, and motivation; and affect and nonhomeostatic eating. Physiological measurements are used to evaluate the levels of cortisol, dehydroepiandrosterone-sulfate (DHEA-S), C-reactive protein, thyroid hormones, blood glucose, glycated hemoglobin, and insulin, as well as allostatic load.

**Results:**

This study has completed participant recruitment (n=60). At the time of study enrollment, the mean age of the participants was almost 47 (SD 10.8) years, and they had a mean BMI of 39.6 (SD 5.31) kg/m^2^. All data are anticipated to be collected by the end of 2023.

**Conclusions:**

We believe that this is the first study to examine changes in body weight and psychological and physiological factors in food-insecure African American women with obesity. This study has significant public health implications because it addresses the cyclic nature of food insecurity to identify underlying mechanisms that can be targeted to mitigate the adverse relationship between food insecurity and obesity and reduce health disparities in minority populations.

**Trial Registration:**

ClinicalTrials.gov NCT05076487; https://clinicaltrials.gov/study/NCT05076487

**International Registered Report Identifier (IRRID):**

DERR1-10.2196/52193

## Introduction

### Background

Food insecurity, or the lack of sufficient food in both quality and quantity for an active and healthy life, is a prevalent public health concern in the United States, with >10% of households reporting food insecurity in 2021 [1,2]. Experiencing food insecurity can lead to adverse chronic health effects, including an increased risk of cardiovascular disease [3-7], type 2 diabetes [7-9], and mortality [10-12]. These associations are important considering chronic disease prevalence among American adults, including >9% with diabetes [13], approximately 33% with hypertension, and another 33% with prehypertension [14]. Because of this, health care spending in the United States is greater in food-insecure adults than in those who are food secure [15].

Food insecurity is linked to poor diet quality [16-19], maladaptive eating behaviors [20-22], and weight gain [23]. Importantly, food insecurity is linked to greater body weight and adiposity among American adults [24-27], with this relationship being the most unequivocal in women [27,28]. The burden of obesity is disparate across population subgroups. By 2030, severe obesity will become the most common BMI category among women, non-Hispanic African American adults, and adults with low income in the United States [29]. Both food insecurity and obesity are more prevalent in African American women than in other racial and ethnic and biological sex groups [30,31]. The prevalence of obesity has been shown to be almost 50% greater in African American women with food insecurity (52.1%) than in White women with food insecurity (26.5%) [32]. These data highlight African American women as a health-disparate population when considering the food insecurity–obesity linkage.

Research on the episodic nature of food insecurity, which is largely understudied in the literature (yet broadly hypothesized), may help elucidate its association with greater body weight (ie, obesity). Most studies that associate food insecurity and obesity are cross-sectional studies [24-28], which is a major limitation, given that food insecurity is considered a *cyclic phenomenon*, with episodes of food adequacy and food shortage [24,33,34]. Those who are food insecure may experience periods when food is more readily available and overconsume during this time, as well as periods of food scarcity that cause restricted food consumption [33,35,36]; for example, the largest federal nutrition assistance program, Supplemental Nutrition Assistance Program (SNAP), provides monthly benefits that are typically dispensed within the first 2 weeks of a month. Given this, food-insecure households may experience food availability in the first weeks of a month and overeat during this time, followed by food scarcity in the latter weeks of the month and constrained food consumption until the beginning of a new month [24,34,35]. Further evidence shows that soup kitchens see an increase in meals served toward the end of the month [37]. This highlights the need for longitudinal studies examining the episodic nature of food insecurity. Furthermore, alternating episodes of food adequacy and food shortage potentially shape body weight response in food-insecure individuals by increasing stress, which has both psychological and physiological manifestations [38].

Stress is a central component in the pathway leading from food insecurity to chronic disease [33,39]. Specifically, “weight gain in food-insecure environments may actually represent a complex network of stress-related reactions” [40]. Experiencing food insecurity can elicit a stress response that leads to the activation of the hypothalamus-pituitary-adrenal (HPA) axis and the release of cortisol [34,41]. Allostasis is the ability to adaptively react to perceived challenges by activating these stress-response processes [42]. Chronic stress and repeated activation of these processes, as brought upon by cycles of food insecurity, can result in maladaptive response, including elevated cortisol levels and higher allostatic load [43]. This has both psychological and physiological costs, leading to increased disease risk [44-46]. Physiological parameters that play a mechanistic role in body weight accretion, such as metabolic and endocrine processes and allostatic load, have not been well assessed in relation to food insecurity. Furthermore, although researchers have found cross-sectional associations between food insecurity and psychological factors, such as poorer cognitive-based decision-making [47,48] and disordered eating [21,22], these associations were not triangulated with body weight. Psychological pathologies and physiological processes may hold insight for elucidating the food insecurity–obesity linkage.

### Objectives

The primary objective of the RESPONSES (Effects of Episodic Food Insecurity on Psychological and Physiological *Responses* in African American Women With Obesity) study is to investigate the association between food insecurity as a stressor and obesity in African American women by assessing longitudinal responses in body weight and psychological and physiological parameters.

## Methods

### Overview of Study Design

**Figure 1:**
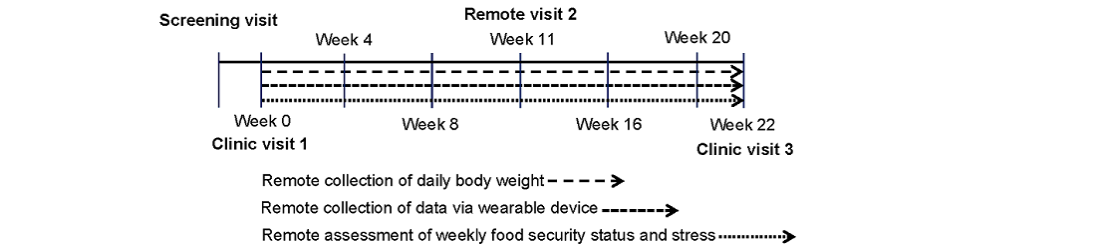
RESPONSES (Effects of Episodic Food Insecurity on Psychological and Physiological Responses in African American Women With Obesity) study design and timeline.

This study is a 22-week longitudinal observation cohort study conducted at Pennington Biomedical Research Center in Baton Rouge, Louisiana. A total of 60 African American women with obesity—30 (50%) who report being food insecure and 30 (50%) who report being food secure at baseline—have been enrolled in the study. Daily body weights are remotely obtained from participants. Wearable devices are also given to participants to wear throughout the duration of the study to collect additional data. Weekly food security status and stress assessments are remotely conducted to ascertain episodes of food insecurity and self-reported stress. Psychological and physiological parameters are measured at the beginning (week 0 at clinic visit 1) and conclusion (week 22 at clinic visit 3) of the study. There is also a midpoint remote assessment of selected psychological parameters (week 11 at remote visit 2). Figure 1 presents the RESPONSES study design and timeline.

The study was registered at ClinicalTrials.gov (NCT05076487) on September 30, 2021.

### Aims

The RESPONSES study will address the following aims.

- Aim 1: the study will longitudinally assess body weight in addition to psychological and physiological parameters in food-insecure and food-secure African American women with obesity. Body weight is collected to assess the change from week 0 to week 22. Psychological parameters (stress; executive function, decision-making, and motivation; and affect and nonhomeostatic eating) are measured via validated questionnaires. Physiological parameters are measured via 24-hour urine (cortisol) and blood (dehydroepiandrosterone-sulfate [DHEA-S], C-reactive protein [CRP], thyroid hormones, blood glucose, glycated hemoglobin, and insulin) samples as well as an allostatic load total score. These parameters are measured at week 0 and week 22 to examine changes in psychological and physiological mechanisms. We hypothesize that food-insecure African American women with obesity will demonstrate increased body weight and changes in psychological and physiological parameters consistent with elevated stress over 22 weeks compared with food-secure African American women with obesity.
- Aim 2: the study will examine associations between changes in body weight and psychological and physiological parameters in food-insecure and food-secure African American women with obesity. We are examining associations between (1) change in body weight collected over 22 weeks and change in psychological and physiological parameters collected 22 weeks apart and (2) changes in psychological and physiological parameters.
- Exploratory aim: the study will longitudinally examine cyclic patterns in body weight, food insecurity, and stress in food-insecure and food-secure African American women with obesity. Body weight is collected daily to assess average weekly change. Food insecurity and stress are assessed weekly via questionnaires. We are examining weekly patterns of body weight, food insecurity, and stress to determine how changes in body weight are related to episodes of food insecurity and stress. Finally, we are assessing mediation analysis of the psychological questionnaires.

### Recruitment and Screening

The target sample is 60 food-secure and food-insecure African American women with obesity; participants are enrolled 1 (food-insecure cohort: n=30, 50%) to 1 (food-secure cohort: n=30, 50%) for an equal number of food-insecure and food-secure participants (1:1) with reported income ≤250% of the federal poverty level. Participants are being recruited from the Greater Baton Rouge, Louisiana, area in designated community locations by trained study staff who assess each prospective participant’s potential eligibility and interest in participating in the study. Community-based recruitment allows for engagement with potential participants in a setting that is familiar and comfortable. Recruitment sites include partnering food pantries, local YMCAs, and other community centers. Community recruitment efforts include, but are not limited to, flyers, presentations, health fairs, and attendance at site-specific community events. Additional recruitment efforts include email blasts, social media posts, and newsletters.

For prescreening, investigators at Pennington Biomedical Research Center use a telephone screener and/or an initial web screener to determine whether potential participants meet basic eligibility criteria. Study staff follow up with potential participants via telephone call to schedule a screening visit to determine eligibility. Ridesharing (eg, Uber and Lyft) is offered to all participants to travel to and from all clinic visits. During the screening visit, potential participants provide written informed consent before the collection of measurements.

Eligibility is assessed using the criteria presented in Textbox 1 (if eligible, participants are enrolled into the study).

**Inclusion and exclusion criteria.**

**Inclusion criteria**

- Willing to provide written informed consent
- Female
- Self-reported race of African American or >1 race in which African American is identified
- Aged 18 to 65 y
- BMI 30 to 50 kg/m^2^ (inclusive)
- Reported income ≤250% of the federal poverty level
- Ability to complete questionnaires in English
- Willing to archive blood and urine samples
- Willing to fast for a minimum of 8 to 10 h before clinic visits

**Exclusion criteria**

- Male
- Self-reported race other than African American or >1 race in which African American is not identified
- Reported income >250% of the federal poverty level
- Given birth within the past 6 mo, currently pregnant, or plans to become pregnant within 6 mo
- Currently breastfeeding
- Currently participating in a weight loss program
- Current use of prescription or over-the-counter medication specifically for weight loss
- Recent weight loss (+5% to −5% weight change in last 6 mo by self-report; if participant is on a prescription medication that impacts weight but meets weight stability criteria [+5% to −5% weight change in last 6 mo by self-report], participant will be deemed eligible)
- Past bariatric surgery or plans for bariatric surgery within 3 mo
- Active cancer or cancer treatment
- Serious digestive disorders that significantly alter metabolism and weight (such as uncontrolled inflammatory bowel disease)
- Current diagnosis of, and active treatment for, alcoholism or other illicit drugs of abuse
- Uncontrolled significant thyroid disorder
- Uncontrolled significant diabetes (fasting blood glucose level >250 mg/dL) or hypertension (blood pressure >180/100 mm Hg)
- Current diagnosis of, or treatment for, heart failure
- Serious psychiatric illness, including bipolar disorder and schizophrenia
- Current or recent diagnosis (within last 5 y) of an eating disorder
- Persons who weigh >380 pounds (172 kg; due to scale requirements)
- Not willing to be recontacted
- Are unable to use devices and applications as required for study participation
- Have another member of the household (same address) who is a participant in the study
- Other conditions that affect metabolism or body weight as determined by the principal investigators and medical investigator
- Presence of any cognitive, psychiatric, behavioral, or medical disorder that, in the opinion of the principal investigators or medical investigator may interfere with study participation

### Outcome Measures and Study Procedures

#### Overview

The primary outcome of the RESPONSES study is the change in *body weight*. The secondary outcomes include *psychological parameters* (stress; executive function, decision-making, and motivation; and affect and nonhomeostatic eating measured via psychological questionnaires) and *physiological parameters* (cortisol, DHEA-S, CRP, thyroid hormones [thyroid-stimulating hormone, free tri-iodothyronine, and free thyroxine], blood glucose, glycated hemoglobin, and insulin levels measured via blood and urine samples, as well as an allostatic load total score). Table 1 summarizes the data collection schedule of study procedures.

**Table 1:** Schedule of RESPONSES (Effects of Episodic Food Insecurity on Psychological and Physiological Responses in African American Women With Obesity) study procedures.

Study procedure		Screening visit	Clinic visit 1 (week 0)	Remote visit 2 (week 11)	Clinic visit 3 (week 22)
Informed consent and HIPAA^a^ authorization		✓			
Health questionnaire and medical history		✓			
Medication inventory		✓			
**Anthropometrics**					
	Height	✓			
	Weight	✓	✓		✓
	Waist and hip circumference	✓	✓		✓
	Bioelectrical impedance analysis	✓	✓		✓
	Blood pressure	✓	✓		✓
	Pulse	✓	✓		✓
**Metabolic or endocrine markers**					
	Fasting blood sample	✓	✓		✓
	24-h urine collection		✓		✓
**Questionnaires**					
	Health literacy	✓			
	Food security status	✓	✓	✓	✓
	Food assistance	✓	✓	✓	✓
	Sociodemographics	✓	✓	✓	✓
	Dietary quality		✓		✓
	Psychological questionnaires^b^		✓	✓^c^	✓

^a^HIPAA: Health Insurance Portability and Accountability Act.

^b^A complete list of psychological and physiological parameters and corresponding questionnaires and measures is presented in Textbox 2.

^c^Excluding International Physical Activity Questionnaire–Long Form, Pittsburgh Sleep Quality Index, and Food Craving Inventory.

**Psychological and physiological parameters assessed in the RESPONSES (Effects of Episodic Food Insecurity on Psychological and Physiological Responses in African American Women With Obesity) study.**

**Psychological parameters and questionnaires**

- Stress
  Perceived Stress Scale (PSS): PSS-10 (clinic visits) and PSS-4 (weekly remote assessment)
- Subjective social status
  MacArthur Scale of Subjective Social Status
- Executive function
  Delay discounting: Kirby 27-item Monetary Choice Questionnaire
- Decision-making
  Grit: 8-item Short Grit Scale
  Future time orientation: Zimbardo Time Perspective Inventory, Consideration of Future Consequences Scale-14
  Financial planning
  Longevity
- Motivation
  Retrospective Visual Analog Scale
- Affect
  Positive and Negative Affect Schedule
- Psychological distress
  6-item Kessler Psychological Distress Scale
- Nonhomeostatic eating
  Food craving: Food Craving Inventory (trait) and Food Craving Questionnaire-State
  Eating disorder: Eating Disorder Diagnostic Scale
  Food addiction: Yale Food Addiction Scale
  Restraint, disinhibition, and hunger: Eating Inventory and Power of Food Scale
- Well-being
  Patient-Reported Outcomes Measurement Information System-43
- Physical activity
  International Physical Activity Questionnaire–Long Form
- Sleep
  Pittsburgh Sleep Quality Index

**Physiological parameters and measures and assessments**

- Stress
  Cortisol: 24-h urine collection
  Dehydroepiandrosterone-sulfate (DHEA-S): fasting blood sample
- Metabolic or endocrine marker
  C-reactive protein, thyroid-stimulating hormone, free tri-iodothyronine, free thyroxine, blood glucose, glycated hemoglobin, and insulin levels: fasting blood sample
- Allostatic load total score
  Cardiovascular health: systolic blood pressure, diastolic blood pressure, pulse, high-density lipoprotein, and total cholesterol levels
  Metabolic markers: albumin levels, waist to hip ratio, and glycated hemoglobin levels
  Immune markers: C-reactive protein levels

#### Screening Visit

All participants undergo a screening visit to determine eligibility. At the screening visit, medical history information is collected, and medications are assessed. Anthropometrics, including height, weight, waist and hip circumference, and blood pressure, are obtained. Height is measured with a standard stadiometer. Measurements are taken without shoes and recorded to the nearest 0.1 cm. For screening purposes, a fasting blood sample was obtained to measure blood glucose, triglycerides, and total cholesterol levels, as well as standard clinical indicators of liver and kidney function.

#### Remote Daily Assessments

##### Body Weight

Body weight is obtained remotely via scales given to participants to take home and weigh themselves at approximately the same time each day. The scales transmit each participant’s body weight data to a secure website to which study staff have access. Participants are given instructions on how to weigh themselves on the scale (eg, flat surface) to obtain accurate data. Ideally, weights are collected in a fasted state. Study staff inspect scale data and contact participants when necessary to ensure compliance with daily weighing.

##### Wearable Device

A wearable device (Fitbit Sense) is provided to each participant at clinic visit 1 to be worn throughout the duration of the study to collect physical activity, sleep, and other device-specific accessible data. The wearable device is used as a monitoring tool, and data are automatically and wirelessly sent to the Fitbit device database. Participants are trained in how to use the device, including how to sync it and charge it regularly.

#### Remote Weekly Assessments

##### Food Security Status

This is assessed via a 2-item food insecurity screener [49,50], which references the previous week, to measure episodes of food insecurity. Weekly food assistance use (eg, SNAP and Special Supplemental Nutrition Program for Women, Infants, and Children benefits, as well as visits to food banks, food pantries, or soup kitchens) and receipt of income from any source (eg, traditional employment and federal financial assistance [Supplemental Security Income, Temporary Assistance for Needy Families, and unemployment benefits]) are also assessed to further augment the assessment of episodic food insecurity.

##### Self-Reported Stress

This is measured using the Perceived Stress Scale-4 (PSS-4).

##### Nonhomeostatic Eating

We also assess nonhomeostatic eating in tandem with weekly assessments of food security status and stress by measuring appetitive drive to consume palatable foods in the absence of hunger via the Power of Food Scale (PFS) [51].

##### Hunger and Fullness

Last, we examine hunger and fullness with 2 questions from the Retrospective Visual Analog Scale [52,53]. These assessments are conducted remotely (eg, via telephone, text, email, or video) on anchor days (eg, Wednesday) for each participant.

#### Clinic Visits 1 and 3

##### Anthropometrics

Fasting *weight* is measured using a standard digital scale. Measurements are taken without shoes and recorded to the nearest 0.1 kg. Measures are taken twice and if not within 0.5 kg are taken a third time. BMI is calculated. *Waist* and *hip circumference* are measured using standardized procedures. Waist circumference is taken from the natural waist. This area is midway between the inferior border of the rib cage and the superior aspect of the iliac crest. The hip circumference is measured at the level of the trochanters, which is usually the maximal extension of the buttocks. Both measures are taken twice and if not within 0.5 cm are taken a third time. Average values are calculated for weight, waist circumference, and hip circumference measurements. Body fat and lean mass are assessed using a body composition analyzer and bioelectrical impedance analysis, which is accurate to within 5% of body fat estimation by dual energy x-ray absorptiometry with a reliability of <1% in variation within itself [54]. *Blood pressure* and *pulse* are taken.

##### Blood Collection

Fasting blood draws are collected. From a chemistry 15 panel, blood glucose, albumin, triglycerides, and cholesterol (total, low-density lipoprotein, and high-density lipoprotein) levels are measured. In addition, DHEA-S, CRP, thyroid-stimulating hormone, free tri-iodothyronine, free thyroxine, and insulin levels are measured.

##### Urinary Cortisol

Urinary cortisol level is obtained from 24-hour urine collections. Participants are provided instructions, a urine hat, and a urine jug. Participants perform the first 24-hour urine collection within 3 days (ideally within 24-48 h) before clinic visit 1. An immunoassay with chemiluminescent detection is used to determine cortisol concentration from urine.

##### Allostatic Load

An *allostatic load total score* is calculated from selected clinic assessments described previously [55].

Textbox 2 presents each psychological and physiological parameter assessed in the RESPONSES study, as well as the relevant questionnaire or metabolic or endocrine assessment used to measure each parameter.

#### Questionnaires

##### Health Literacy

The Rapid Estimate of Adult Literacy in Medicine–Short Form (REALM-SF) is used to measure health literacy in participants. This assessment is a standardized series of 7 words. The score (0-7) provides an assessment of how well a participant will be able to understand the additional questionnaires. Participants scoring 0 to 3 on the REALM-SF are aided by study staff in completing the other questionnaires. Patients scoring 4 to 7 on the REALM-SF complete the other questionnaires on their own but are offered assistance as needed.

##### Food Security Status

This is measured using a modified version of the 6-item Food Security Scale [56,57]. This scale is a well-validated measure of food security developed by researchers at the National Center for Health Statistics. Two or more affirmative answers indicate food insecurity.

##### Food Assistance

Participants will be asked about their use of federal food assistance (eg, SNAP [food stamps] and Special Supplemental Nutrition Program for Women, Infants, and Children) and other forms of nonfederal food assistance (eg, visits to food pantries and soup kitchens).

##### Sociodemographics

Participants complete a questionnaire that assesses age (date of birth), race, ethnicity, sex, marital status, the highest level of education completed, annual household income, the number of people in the household, occupation, employment status, home street address (including city and zip code), sources of financial support, dependent status, insurance and health care coverage, and smoking status.

##### Dietary Quality

A screener that assesses dietary fat, fruit, vegetable, and alcohol intake is completed by participants. The screener contains scales from several sources. The National Cancer Institute fat screener estimates the percentage of energy from fat by asking participants to report the frequency of consuming specific foods over the past 12 months [58,59]. A standard 7-item fruit and vegetable screener developed by the National Cancer Institute and the National 5 a Day Program asks how often fruit and vegetables were consumed in the past month [60,61]. Three questions related to the frequency of alcohol (beer, wine, and hard liquor) intake were adapted from the Brief Questionnaire to Assess Habitual Beverage Intake [62].

#### Psychological Questionnaires

Participants also complete multiple self-report questionnaires to measure psychological constructs related to stress; executive function, decision-making, and motivation; and affect and nonhomeostatic eating, as well as overall well-being, physical activity, and sleep. All questionnaires are reliable and valid as described in the following subsections.

##### Perceived Stress Scale

The Perceived Stress Scale-10 (PSS-10) is the most widely used psychological instrument to measure the perception of stress [63]. This questionnaire includes 10 items that measure the degree to which situations in one’s life are appraised as stressful. The PSS-4 is a shortened version of the PSS-10 designed for telephone interviews. Items are designed to tap how unpredictable, uncontrollable, and overloaded respondents find their lives. Higher scores on the PSS-10 and PSS-4 indicate greater stress.

##### Subjective Social Status

This construct indicates one’s self-perceived social position in American society and will be measured using the MacArthur Scale of Subjective Social Status [64]. This scale presents a *ladder* and asks participants to select a rung on which they feel they stand compared with other people in the United States. Scores range from 1 to 10, with higher scores representing higher subjective social status.

##### Kirby 27-Item Monetary Choice Questionnaire

Delay discounting is a bias toward smaller immediate rewards versus larger delayed rewards [65] and will be assessed via the Kirby 27-item Monetary Choice Questionnaire [66]. This questionnaire presents participants with a set of choices between smaller immediate monetary rewards and larger delayed monetary rewards. Participants who discount the value of the delayed rewards more steeply are considered to be more impulsive [66].

##### 8-Item Short Grit Scale

Grit is a measure of trait-level perseverance and passion for long-term goals and will be assessed using the 8-item Short Grit Scale [67]. Scores range from 1 (not at all gritty) to 5 (extremely gritty).

##### Zimbardo Time Perspective Inventory–Future Scale

Future time perspective is a comprehensive assessment of one’s orientation toward the future [47,68]. The *future* scale of the Zimbardo Time Perspective Inventory measures psychological orientation toward the future [69]. This scale comprises 13 statements with Likert-type responses. Higher scores indicate greater future time perspective.

##### Consideration of Future Consequences Scale

This scale is a valid and reliable 14-item measure of the extent to which a participant considers future outcomes in relation to current behaviors. The measure assesses two subscales—(1) consideration of immediate consequences and (2) consideration of future consequences—to assess the extent to which a participant feels that they may be influenced by an abstract consequence. The participant indicates how much they agree with the statement on a scale from 1 (not at all like you) to 7 (very much like you) [70-72]. Higher scores indicate greater consideration of future consequences.

##### Financial Planning

Participants will be asked a single question to assess the time period considered for financial planning: “In planning your, or your family’s, saving and spending, which of the following time periods is more important to you and your partner, if you have one?” with the following response options: no planning, day to day, the next few weeks, the next few months, the next year, the next few years, the next 5 to 10 years, longer than 10 years [73,74]. Higher values indicate greater future time perspective.

##### Longevity

Participants’ subjective appraisal of longevity will also be assessed via a single question: “What do you think are the chances you will live to age 75 or more (where 0 means there is not a chance you will live to 75 or more, and 100 means you will definitely live to 75 or more)?” [73,74]. Higher values indicate greater future time perspective.

##### Retrospective Visual Analog Scale

This scale contains 9 items to measure the average ratings of appetitive sensations that participants experienced over the past week. This method of collecting visual analog scale data has been found to be consistent with daily assessments of satiety [52], and support has been found for the reliability and validity of the visual analog scale for measuring subjective states related to energy intake [53].

##### Positive and Negative Affect Schedule

This is a self-report measure that is made up of 2 mood scales, 1 measuring positive affect and 1 measuring negative affect [75]. It has 10 items measuring positive affect and 10 items measuring negative affect on a 5-point Likert scale ranging from 1 (very slightly or not at all) to 5 (extremely). Scores can range from 10 to 50 on each scale, with higher scores representing higher levels of positive affect and lower scores representing lower levels of negative affect.

##### Psychological Distress

The Kessler Psychological Distress Scale (short version) is a 6-item self-report measure of psychological distress [76]. Respondents indicate how often they have had 6 different feelings or experiences during the past 30 days using a 5-point Likert scale: 4 (all of the time), 3 (most of the time), 2 (some of the time), 1 (a little of the time), and 0 (none of the time). The feelings and experiences are the following: *nervous*, *hopeless*, *restless or fidgety*, *so depressed that nothing could cheer you up*, *that everything was an effort*, and *worthless*. The score ranges from 0 to 24, with higher scores indicating greater psychological distress.

##### Food Craving Inventory

This is a 33-item measure of food craving [77,78] and assesses the frequency with which an individual experiences a craving for a particular food. The measure consists of 5 empirically derived factors: high fats, sweets, carbohydrates and starches, fruits and vegetables, and fast-food fats.

##### Food Craving Questionnaire-State

This questionnaire was developed to measure state (at the moment) craving for food using 15 items [79]. The reliability and validity of the state version of the Food Craving Questionnaire have been established.

##### Eating Disorder Diagnostic Scale

This brief scale contains 18 self-report items to assess eating disorder pathology and has been shown to be reliable and valid [80]. This measure can diagnose anorexia nervosa, bulimia nervosa, and binge eating disorder.

##### Yale Food Addiction Scale

This is a 27-item self-report questionnaire [81] used to identify individuals showing tendencies for addictive-like behaviors toward certain types of foods, such as those high in fat or sugar. The measure also allows participants to subjectively identify specific problem foods.

##### Eating Inventory

The Three-Factor Eating Inventory is designed to measure different dimensions of eating behavior. Three factor-analyzed subscales (cognitive restraint, disinhibition, and hunger) are derived from the questionnaire [82]. The 51-item questionnaire is divided into 2 parts: part I is composed of 36 true-false items, and part II is composed of 15 rating scale items.

##### Power of Food Survey

The PFS measures loss-of-control, or nonhomeostatic, eating. The PFS comprises 15 items that use a 5-point Likert scale to assess the intensity of desire to consume delicious food when not hungry [51]. Higher scores indicate greater hedonic hunger.

##### Patient-Reported Outcomes Measurement Information System-43

The Patient-Reported Outcomes Measurement Information System (PROMIS) is a system of highly reliable and precise measures of patient-reported health status for physical, mental, and social well-being [83,84]. The PROMIS-43 is a 43-item health-related quality-of-life questionnaire that includes questions covering the health-related domains of physical function, anxiety, depression, fatigue, sleep disturbance, ability to participate in social roles and activities, pain interference, and pain intensity.

##### International Physical Activity Questionnaire–Long Form

This is a 7-day recall of the intensity of physical activity and has acceptable reliability [85]. The results are reported as a continuous measure in median metabolic equivalent of task minutes per week.

##### Pittsburgh Sleep Quality Index

This is a self-rated questionnaire to assess sleep quality and disturbance over a 1-month time interval [86]. A total of 19 individual items generate 7 *component* scores: subjective sleep quality, sleep latency, sleep duration, habitual sleep efficiency, sleep disturbances, the use of sleeping medication, and daytime dysfunction. The sum of the scores for these 7 components yields 1 global score. Acceptable measures of internal homogeneity, consistency (test-retest reliability), and validity have been obtained [86].

##### Resource Packet

Given the focus on women who experience food insecurity, a resource packet was developed for the RESPONSES study that identifies community resources and services available to participants who may face resource deficits that compromise their overall health. Importantly, participants may identify multiple resource deficits beyond food insecurity during their participation in the study. The resource packet includes relevant community resources and services that address a range of health-related issues and concerns, including food insecurity, medical and dental care, mental health, substance abuse, and domestic violence. The resource packet is given to all participants regardless of study completion or food security status.

### Management of Study Data

Portions of RESPONSES study data are collected and managed using Research Electronic Data Capture (REDCap; Vanderbilt University) hosted at Pennington Biomedical Research Center. REDCap is a secure Health Insurance Portability and Accountability Act–compliant web-based application designed to support data capture for research studies, providing (1) an intuitive interface for validated data entry, (2) audit trails for tracking data manipulation and export procedures, (3) automated export procedures for seamless data downloads to common statistical packages, and (4) procedures for importing data from external sources [87].

### Sample Size and Statistical Power

Change in body weight was used to compute a minimum sample size for at least 80% power to examine response to episodic food insecurity longitudinally in African American women with obesity. Specifically, the power analysis is based on a study by Wilde and Peterman [23], which used nationally representative data to examine change in body weight over a 1-year period by food security status in women and men. We chose these data on body weight because we expect this measure to be responsive to episodic food insecurity. Wilde and Peterman [23] found that food-secure women had a 10.4% chance of gaining between 2.27 kg and 4.54 kg and a 20.7% chance of gaining >4.54 kg during a 1-year period. Food-insecure women had a 13% chance and a 31.7% chance of gaining between 2.27 kg and 4.54 kg and >4.54 kg, respectively. Using these estimates, an expected change over 22 weeks in the food-secure group is 0.54 kg, and an expected change in the food-insecure group is 0.80 kg. An SD of 22-week change in body weight, 0.24 kg [88], was increased to 0.30 kg to account for higher variability and ensure that the proposed study is well powered. Thus, with 60 participants, the study is well powered (>80%) to detect differences in body weight changes for aim 1. Furthermore, this study is also powered to detect a correlation as small as 0.40 for aim 2.

### Planned Statistical Analysis

All available daily body weights will be used for analyses. For aim 1, body weight and psychological and physiological parameters will be analyzed using a mixed effects model with time, food security status, baseline BMI, and age. Models will be checked for both normality and heterogeneity of variance in potential subgroups (eg, age and BMI category). Least squares means and tests for differences by food security group will be the primary results. The analysis of aim 2 will focus on correlations among the outcomes from aim 1. Both Spearman and Pearson correlation coefficients will be used to determine the pairwise relationships among outcomes. Depending on the collinearity among outcomes, factor analysis will also be used to explore these relationships. Analysis of the exploratory aim will determine whether the weekly patterns of change in body weight, food security status, and perceived stress are different by food security group and how well these measures are associated with each other over time.

For aim 1, mixed effects models will be used to model psychological and physiological parameters. Furthermore, a linear mixed effects model will be used to model the daily weight over time. In addition to overall changes in body weight between the groups adjusted for BMI, age, and other baseline covariates, smaller temporal changes (ie, weekly) will also be a focus. Modeling these smaller changes should account for the increased fluctuations in the weight of the food-insecure women.

For aim 2, mixed effects models will assess how daily weight change is associated with changes in psychological and physiological parameters. Initial weight, overall weight change, and weekly weight change will all be considered as predictors in addition to more exploratory weight variables, such as the number of days significantly above or below starting weight or average absolute daily weight changes. These variables will be interacted with food security status (group) to determine whether the weight changes have greater effect on food-insecure women.

Analysis of the exploratory aim will determine whether the weekly patterns of change in body weight, food security status, and perceived stress are different by food security group and how well these measures are associated with each other over time. The first part of this analysis will use a mixed effects model, testing food security group differences at each week, whereas the second part will test correlations. Mediation analyses will be applied using both structural mean models and principal stratification [89]. Weekly food assistance use may be used for subgroup analyses to explore how food assistance use influences body weight.

### Ethical Considerations

The Pennington Biomedical Research Center Institutional Review Board approved the study protocol (2021-022-PBRC), and all patients provided written informed consent. All attempts are made to maintain participant privacy and confidentiality. Safeguards such as password-protected computers and networks are in place to limit access to participant data. Study data are labeled with a unique series of letters and numbers. Pennington Biomedical Research Center stores data with this unique identifier. Participants receive up to US $300 for completed participation in the study. This includes compensation of US $50 for completion of visit 1, US $50 for completion of visit 2, and US $125 for completion of visit 3. Participants also receive the wearable device upon completion of the study.

## Results

### Overview

**Figure 2:**
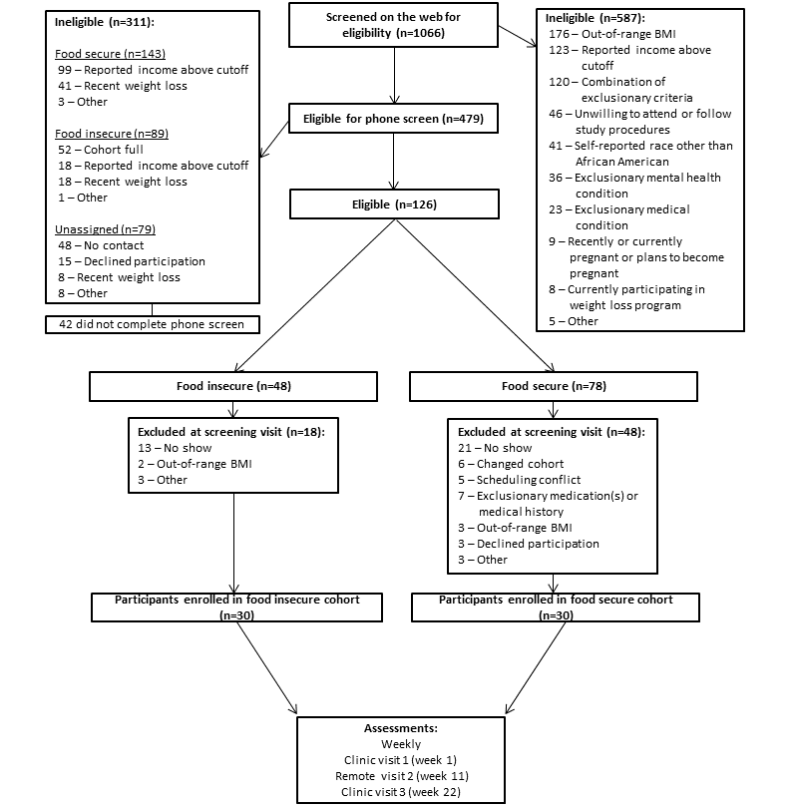
RESPONSES (Effects of Episodic Food Insecurity on Psychological and Physiological Responses in African American Women With Obesity) participant flow diagram.

The RESPONSES study has completed participant recruitment (n=60). The flow of participants from screening to enrollment is shown in Figure 2. A total of 1066 potential participants were screened via a web screener for enrollment, and 587 (55.07%) were ineligible for study participation; the most common exclusionary criteria were out-of-range BMI (n=176, 30%) and having a reported income above the cutoff (n=123, 21%). Of the 479 potential participants who were screened via a telephone call, 311 (64.9%) were ineligible owing to exclusionary criteria. Of the 126 potential participants who were eligible after the telephone screener, 48 (38.1%) were food insecure, and 78 (61.9%) were food secure. At screening visits, of the 126 potential participants, a combined 66 (52.4%) were excluded from study participation to reach the target sample size of 60 (47.6%; food-insecure cohort: n=30, 50%; food-secure cohort: n=30, 50%). All data are anticipated to be collected by the end of 2023.

### Participant Characteristics

Table 2 presents the baseline characteristics of the participants enrolled in the RESPONSES study. On average, participants were aged almost 47 (SD 10.8) years with a mean BMI of 39.6 (SD 5.31) kg/m^2^ at the time of study enrollment. Regarding marital status, 76% (45/59) of the participants were divorced, separated, widowed, or never married. In terms of education level, 58% (35/60) of the participants had a high school diploma, General Educational Development Test certificate, or 1 to 3 years of college. Regarding income, 31% (17/55) of the participants reported an annual income of <US $30,000, and 24% (13/55) reported an annual income of >US $50,000. In terms of employment status, 58% (33/57) of the participants reported being employed full time. The mean poverty-to-income ratio (PIR) among all participants was 1.96 (SD 0.83), whereas the food-insecure cohort reported an average PIR of 2.03 (SD 0.79), and the food-secure cohort reported an average PIR of 1.88 (SD 0.89).

**Table 2:** Baseline characteristics of participants enrolled in the RESPONSES (Effects of Episodic Food Insecurity on Psychological and Physiological Responses in African American Women With Obesity) study.

Characteristics		Food-insecure cohort	Food-secure cohort	Overall
**Age (y)**				
	Participants, n	30	30	60
	Value, mean (SD)	46.93 (10.45)	46.47 (11.32)	46.7 (10.8)
**Weight (kg)**				
	Participants, n	30	30	60
	Value, mean (SD)	106.42 (17.13)	105.82 (14.94)	106.12 (15.94)
**BMI (kg/m^2^)**				
	Participants, n	30	30	60
	Value, mean (SD)	39.31 (5.4)	39.95 (5.29)	39.63 (5.31)
**Waist circumference (cm)**				
	Participants, n	29	30	59
	Value, mean (SD)	114.49 (11.33)	112.9 (10.46)	113.68 (10.83)
**Hip circumference (cm)**				
	Participants, n	29	29	58
	Value, mean (SD)	126.69 (13.87)	126.98 (10.7)	126.84 (12.28)
**Waist to hip ratio**				
	Participants, n	29	29	58
	Value, mean (SD)	0.91 (0.07)	0.89 (0.08)	0.9 (0.07)
**Pulse**				
	Participants, n	29	30	59
	Value, mean (SD)	66.55 (9.65)	67.33 (9.39)	66.95 (9.44)
**Marital status, n/N (%)**				
	Married	5/30 (17)	9/29 (31)	14/59 (24)
	Divorced, separated, widowed, or never married	25/30 (83)	20 (69)	45/59 (76)
**Education, n/N (%)**				
	High school diploma, GED^a^ certificate, or 1-3 y college	20/30 (67)	15/30 (50)	35/60 (58)
	Bachelor’s degree or postgraduate degree	10/30 (33)	15/30 (50)	25/60 (42)
**Annual income (US $), n/N (%)**				
	<30,000	8/29 (28)	9/26 (35)	17/55 (31)
	30,000-39,000	8/29 (28)	5/26 (19)	13/55 (24)
	40,000-49,999	9/29 (31)	3/26 (12)	12/55 (22)
	>50,000	4/29 (14)	9/26 (35)	13/55 (27)
**Household size**				
	Participants, n	29	29	58
	Value, mean (SD)	2.48 (1.48)	3 (1.54)	2.74 (1.52)
**Employment status, n/N (%)**				
	Employed full time (40 h/wk)	16/28 (57)	17/29 (59)	33/57 (58)
	Employed part time	3/28 (11)	3/29 (10)	6/57 (11)
	Unemployed	2/28 (7)	1/29 (3)	3/57 (5)
	Retired	0/28 (0)	4/29 (14)	4/57 (7)
	Medical disability	2/28 (7)	1/29 (3)	3/57 (5)
	Student	0/28 (0)	2/29 (7)	2/57 (4)
	Self-employed, homemaker, or other	3/28 (11)	1/29 (3)	4/57 (7)
	Other	2/28 (7)	0/29 (0)	2/57 (4)
**Financial support, n/N (%)**				
	Family	2/16 (13)	2/10 (20)	4/26 (15)
	Employment	8/16 (50)	5/10 (50)	13/26 (50)
	Government	2/16 (13)	1/10 (10)	3/26 (12)
	Scholarships	2/16 (13)	1/10 (10)	3/26 (12)
	Loans	0/16 (0)	0/10 (0)	0/26 (0)
	Other	2/16 (13)	1/10 (10)	3/26 (12)
**Dependent, n/N (%)**				
	Yes	2/30 (7)	0/30 (0)	2/60 (3)
	No	28/30 (93)	30/30 (100)	58/60 (97)
**Insurance, n/N (%)**				
	None	1/30 (3)	0/30 (0)	1/60 (2)
	Private	16/30 (53)	17/30 (57)	33/60 (55)
	Medicare	4/30 (13)	5/30 (17)	9/60 (15)
	Medicaid	9/30 (30)	8/30 (27)	17/60 (28)
	Use parents’ or legal guardians’ insurance coverage	0/30 (0)	0/30 (0)	0/60 (0)
**Poverty-to-income ratio**				
	Participants, n	28	26	54
	Value, mean (SD)	2.03 (0.79)	1.88 (0.89)	1.96 (0.83)
**Creatinine, n (mean, SD)**				
	Participants, n	30	30	60
	Value, mean (SD)	0.82 (0.21)	0.86 (0.18)	0.84 (0.19)
**Potassium**				
	Participants, n	30	30	60
	Value, mean (SD)	4.23 (0.39)	4.24 (0.37)	4.24 (0.37)
**Uric acid**				
	Participants, n	30	30	60
	Value, mean (SD)	5.49 (1.03)	5.51 (1.06)	5.5 (1.04)
**Calcium**				
	Participants, n	30	30	60
	Value, mean (SD)	9.4 (0.36)	9.39 (0.36)	9.4 (0.36)
**Magnesium**				
	Participants, n	30	30	60
	Value, mean (SD)	2.01 (0.16)	2 (0.21)	2.01 (0.19)
**CPK^b^**				
	Participants, n	30	30	60
	Value, mean (SD)	134.6 (110.47)	148.3 (97.23)	141.45 (103.41)
**ALT^c^**				
	Participants, n	30	30	60
	Value, mean (SD)	19.33 (6.87)	17.33 (0.87)	18.33 (6.42)
**ALK^d^**				
	Participants, n	30	30	60
	Value, mean (SD)	77.93 (27.01)	69.13 (19.78)	73.53 (23.89)
**Iron**				
	Participants, n	30	30	60
	Value, mean (SD)	66.13 (23.08)	62.5 (24.41)	64.32 (23.62)
**Type 1 diabetes, n/N (%)**				
	Yes	2/30 (7)	1/30 (3)	3/30 (5)
	No	28/30 (93)	29/30 (97)	57/30 (95)
**Type 2 diabetes, n/N (%)**				
	Yes	4/30 (13)	8/30 (27)	12/60 (20)
	No	26/30 (87)	22/30 (73)	48/60 (80)
**High blood pressure, n/N (%)**				
	Yes	13/27 (48)	12/28 (43)	25/55 (45)
	No	14/27 (52)	16/28 (57)	30/55 (55)
**High cholesterol level, n/N (%)**				
	Yes	9/26 (35)	4/28 (14)	13/54 (24)
	No	17/26 (65)	24/28 (86)	41/54 (76)

^a^GED: General Educational Development Test.

^b^CPK: creatine phosphokinase.

^c^ALT: alanine transaminase.

^d^ALK: alkaline phosphatase.

## Discussion

### Summary

The RESPONSES study is investigating the association between food insecurity and obesity in African American women by measuring the episodic nature of food insecurity, related stress, and the role of psychological and physiological mechanisms. We have completed participant enrollment with 60 African American women (food-insecure cohort: n=30, 50%; food-secure cohort: n=30, 50%). The mean age of participants at enrollment was almost 47 (SD 10.8) years, and they had a mean BMI of 39.6 (SD 5.31) kg/m^2^. Once data collection is complete by the end of 2023, we will fulfill the study aims to (1) longitudinally assess body weight in addition to psychological and physiological parameters in food-insecure and food-secure African American women with obesity, to test our hypothesis that food-insecure African American women with obesity will demonstrate increased body weight and changes in psychological and physiological parameters consistent with elevated stress compared with food-secure African American women with obesity; (2) examine associations between changes in body weight and psychological and physiological parameters in food-insecure and food-secure African American women with obesity; and (3) longitudinally examine cyclic patterns in body weight, food insecurity, and stress in food-insecure and food-secure African American women with obesity and carry out a mediation analysis using the results from the psychological questionnaires.

The significance of the RESPONSES study is evidenced by a number of factors, including (1) an explicit focus on health disparities driven by both sex and race or ethnicity as evidenced in African American women, who experience a greater prevalence of both food insecurity and obesity; (2) the assessment of episodic food insecurity via longitudinal observational data collection; and (3) the triangulation of body weight and psychological and physiological parameters in the context of episodic food insecurity as a stressor. These points of significance directly relate to the *Strategic Plan for NIH Nutrition Research* by addressing the multiple recommendations highlighted in the position paper on food insecurity and obesity by Brown et al [90]. This study explicitly acknowledges the *episodic nature* of food insecurity by using a longitudinal study design to examine how the cyclic nature of food insecurity relates to body weight. Furthermore, this study will investigate the mechanisms underlying the linkage between food insecurity and obesity by focusing on a range of relevant psychological and physiological factors. Examining the interplay between psychological and physiological factors may be important to understanding the disparate rates of obesity-related comorbidities (eg, diabetes and hypertension) in African American women. Finally, the results from the RESPONSES study will hold important considerations for the assessment of body weight and psychological and physiological parameters in studies, wherein the assessment of these markers might need to consider corresponding episodes of food insecurity.

The RESPONSES study uses a longitudinal observational cohort design. Given the study objective, it is important not to intervene; thus, this study would not be possible with a randomized controlled trial design. Interventions should occur after a thorough understanding of the episodic nature of food insecurity and its impact on body weight and psychological and physiological processes. We recognize that only screening for dietary quality and not obtaining food intake data are study limitations, but this aspect of food insecurity (ie, energy-dense and nutrient-poor diet) is very well established in the literature, and body weight can serve as a proxy for food intake [19,20,91]. We are also measuring more tertiary constructs (eg, physical activity) that could be measured via more rigorous methods in future studies, such as energy intake via dietary recall or food pictures and energy expenditure via metabolic chamber or other methodology (eg, doubly labeled water and metabolic cart) to better assess energy metabolism.

### Conclusions

To our knowledge, the RESPONSES study will be the first to examine changes in body weight and psychological and physiological factors in food-insecure African American women with obesity during episodes of food insecurity. The findings from this study will provide a range of novel data with significant public health implications by addressing the cyclic nature of food insecurity to identify underlying mechanisms that can be targeted to mitigate the adverse relationship between food insecurity and obesity and reduce health disparities in racially minoritized populations.

## Abbreviations

CRP: C-reactive protein
DHEA-S: dehydroepiandrosterone-sulfate
HPA: hypothalamus-pituitary-adrenal
PFS: Power of Food Scale
PIR: poverty-to-income ratio
PROMIS: Patient-Reported Outcomes Measurement Information System
PSS-10: Perceived Stress Scale-10
PSS-4: Perceived Stress Scale-4
REALM-SF: Rapid Estimate of Adult Literacy in Medicine–Short Form
REDCap: Research Electronic Data Capture
RESPONSES: Effects of Episodic Food Insecurity on Psychological and Physiological Responses in African American Women With Obesity
SNAP: Supplemental Nutrition Assistance Program

## Data Availability

RESPONSES (Effects of Episodic Food Insecurity on Psychological and Physiological Responses in African American Women With Obesity) study data will be a deidentified participant-level data set that will include all data points necessary to validate the anticipated research findings. A cleaned, deidentified data set will be encrypted and stored on a secure server at Pennington Biomedical Research Center. At study completion, data will be made available to users via request from the Pennington Nutrition Obesity Data Repository. The mission of the Pennington Nutrition Obesity Research Centers Biorepository is to advance the knowledge of obesity, nutrition, and human health through the sharing of deidentified data and biospecimens collected from human participant research at Pennington Biomedical Research Center.
